# Modulation of Oxidative Stress and Neuroinflammation by Cannabidiol (CBD): Promising Targets for the Treatment of Alzheimer’s Disease

**DOI:** 10.3390/cimb46050266

**Published:** 2024-05-06

**Authors:** Jordan P. Hickey, Andrila E. Collins, Mackayla L. Nelson, Helen Chen, Bettina E. Kalisch

**Affiliations:** Department of Biomedical Sciences and Collaborative Specialization in Neuroscience Program, University of Guelph, Guelph, ON N1G 2W1, Canada; jhicke01@uoguelph.ca (J.P.H.); andrila@uoguelph.ca (A.E.C.); mackayla@uoguelph.ca (M.L.N.); hchen16@uoguelph.ca (H.C.)

**Keywords:** cannabidiol, CBD, Alzheimer’s disease, neuroinflammation, oxidative stress, anti-inflammatory properties, antioxidant properties, endocannabinoid system, review

## Abstract

Alzheimer’s disease (AD) is a progressive neurodegenerative disease and the most common form of dementia globally. Although the direct cause of AD remains under debate, neuroinflammation and oxidative stress are critical components in its pathogenesis and progression. As a result, compounds like cannabidiol (CBD) are being increasingly investigated for their ability to provide antioxidant and anti-inflammatory neuroprotection. CBD is the primary non-psychotropic phytocannabinoid derived from *Cannabis sativa.* It has been found to provide beneficial outcomes in a variety of medical conditions and is gaining increasing attention for its potential therapeutic application in AD. CBD is not psychoactive and its lipophilic nature allows its rapid distribution throughout the body, including across the blood–brain barrier (BBB). CBD also possesses anti-inflammatory, antioxidant, and neuroprotective properties, making it a viable candidate for AD treatment. This review outlines CBD’s mechanism of action, the role of oxidative stress and neuroinflammation in AD, and the effectiveness and limitations of CBD in preclinical models of AD.

## 1. Introduction

Cannabinoids, found in the *Cannabis sativa* plant, are a class of biological compounds that have gained increasing attention for their potential therapeutic effects and applications in treating multiple health conditions, including Alzheimer’s disease (AD) [1,2]. Support for the therapeutic use of cannabis or cannabis extracts comes primarily from preclinical studies that demonstrate the ability of cannabinoids to target multiple processes reported to underlie the development and progression of AD pathology.

AD is a neurodegenerative disease that is characterized by progressive impairments in learning, memory, and cognition [3,4,5]. Although the precise underlying cause of AD remains elusive, research suggests that oxidative stress and neuroinflammation play crucial roles in its pathogenesis and progression [3,4,5]. Oxidative stress results from a disrupted balance in the pro-oxidant/antioxidant systems, leading to the production of reactive oxygen species (ROS) and free radicals [4,6,7]. In AD, along with disturbed metal metabolism, oxidative stress causes damage to cellular components, such DNA, proteins, and lipids, and contributes to inflammation, neuronal dysfunction, and neuronal loss [4,6,7]. Similarly, neuroinflammation in AD, initiated by immune responses from microglia, exacerbates neurodegeneration through the chronic activation of inflammatory cascades that contribute to disease progression [8,9]. Additionally, there is much evidence supporting the interplay between neuroinflammation, oxidative stress, and the production and accumulation of amyloid-beta (Aβ) plaques and neurofibrillary tau tangles (NFTs), which are the well-known pathological hallmarks of AD [7,10]. This complex relationship creates a self-perpetuating cycle, further accelerating the degenerative process in AD [7,10]. As such, targeting oxidative stress and neuroinflammation represents an important strategy for the treatment of AD.

Cannabidiol (CBD) is one of the most widely studied cannabinoids and has emerged as a potential therapeutic for AD due to its antioxidant and anti-inflammatory properties as well as its non-psychoactive nature [1,2,11,12,13]. CBD plays a role in reducing ROS production and increasing the level of and supporting endogenous antioxidant activities, thus minimizing oxidative damage to cells [13]. Additionally, CBD has been shown to inhibit the migration of activated microglial cells, suppress the expression and activity of proinflammatory mediators, and induce anti-inflammatory cytokines, thereby mitigating the harmful effects of chronic neuroinflammation [11,12,13]. CBD’s ability to address both the neuroinflammation and the oxidative stress associated with AD makes it a promising therapeutic candidate for slowing the progression of the condition.

**Figure 1:**
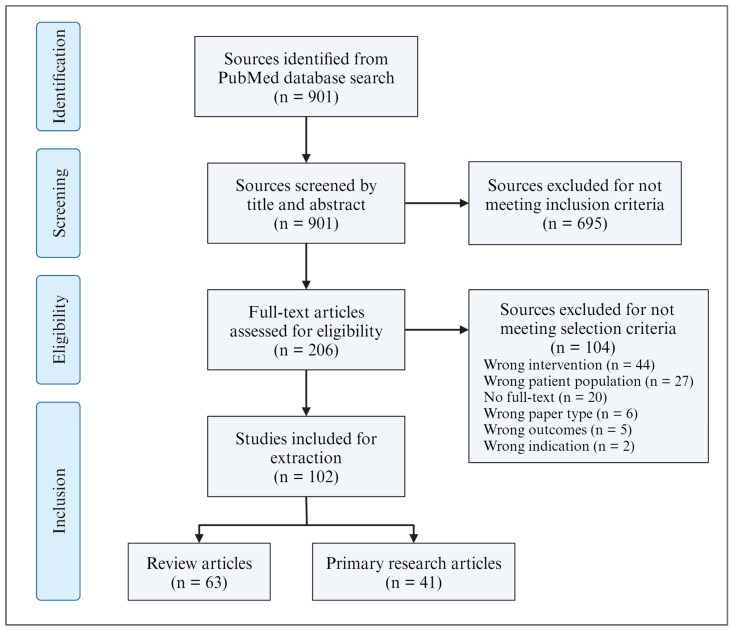
Flowchart representing the article identification and screening processes. Created using BioRender.com.

This review explores the therapeutic potential of CBD by evaluating its effects in preclinical in vitro and in vivo models of AD and the mechanisms underlying its anti-inflammatory and antioxidant properties. Articles were identified by conducting a comprehensive search on PubMed (1924–29 November 2023). Using Covidence, an article screening and data collection software, sources were assessed by two independent reviewers for inclusion eligibility, and in the case of a conflict, a third reviewer was required to reach a consensus. Articles included in the final review were from English-language peer-reviewed journals and (1) focused primarily on AD or neurodegenerative diseases with shared mechanisms, (2) utilized CBD as a primary treatment or therapeutic of study, and (3) assessed AD-like pathology, behaviour, and anti-inflammatory and/or antioxidant outcomes (Figure 1).

## 2. General Features of Cannabidiol

In recent years, exogenous cannabinoids, derived from *Cannabis sativa*, have gained significant attention in biomedical research, particularly Δ-9-tetrahydrocannabinol (THC) and CBD, the two predominant phytocannabinoids [2,14]. THC and CBD are exogenous lipophilic signalling molecules, the former being the main psychotropic substituent of cannabis [2,14]. The behavioural and analgesic effects of cannabis and cannabis extracts prompted investigation into the mechanism of action of cannabinoids in mammalian physiology, resulting in the discovery of the endocannabinoid system (ECS) and a better understanding of the pharmacological properties and therapeutic potential of CBD [2,14].

CBD has been demonstrated to exhibit a range of anxiolytic, antipsychotic, anticonvulsive, antinausea, anti-inflammatory, anti-rheumatoid arthritic, and antioxidant effects [15,16,17]. Most notably, CBD exhibits strong anti-epileptic properties, with the US Food and Drug Administration (FDA) approving Epidiolex^®^ for the treatment of refractory epilepsy in 2018 [16]. The various complex pharmacological actions of CBD make it an interesting potential therapeutic for a variety of pathologies, including movement disorders, chronic pain, psychiatric disorders, cancer, and neurodegenerative diseases, such as Parkinson’s disease (PD) and AD [15,16,17]. These potential health benefits are likely due to CBD’s interaction with the ECS.

### 2.1. The Endocannabinoid System

The ECS is a highly expressed complex of receptors and ligands throughout the central and peripheral nervous systems (CNS and PNS, respectively). This system consists of the cannabinoid type 1 (CB1) and 2 (CB2) receptors, which are member of the superfamily of G-protein coupled receptors (GPCRs) [2,14,18]. The CB1 receptor is ubiquitously expressed throughout the CNS, serving as the brain’s most abundant GPCR, but it is also expressed in a variety of peripheral tissues [18]. CB1 receptors are primarily found on presynaptic GABAergic interneurons and to a lesser extent on other neuronal subtypes such as glutamatergic and cholinergic neurons [18,19,20]. Conversely, the CB2 receptor was originally believed to only be expressed on immune cells in the periphery, however, more recently it has been reported to be present in the CNS, primarily on microglial cells [18,21,22,23,24].

**Figure 2:**
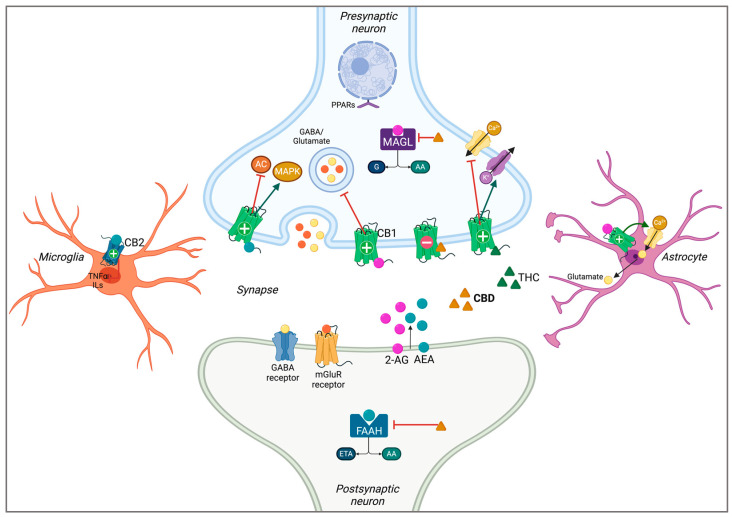
Schematic representation of the endocannabinoid system in the CNS. Postsynaptically synthesized endocannabinoids (eCBs) 2-AG (pink) and AEA (blue) are retrograde signalling molecules that bind and activate CB1 receptors. The psychotropic phytocannabinoid THC (green) is a CB1 agonist, while CBD (yellow) is a negative allosteric modulator (negative symbol) of CB1, inversely agonizing the receptor. CBD also inhibits the enzymes involved in eCB degradation, FAAH and MAGL. CB1 activation promotes astrocytic Ca^2+^ influx and presynaptic MAPK and GIRK activity, and inhibits AC activity, voltage-gated Ca^2+^ channels, and neurotransmitter release from the presynaptic neuron. CB2 activation in microglia can result in the inhibition of inflammatory cytokines TNFɑ and ILs [2,15,19,31,32,33]. CBD: cannabidiol; THC: Δ-9-tetrahydrocannabinol; 2-AG: 2-arachidonoylglycerol; AEA: anandamide; CB1/2: cannabinoid type receptors 1 and 2; MAGL: monoacylglycerol lipase; FAAH: fatty acid amide hydrolase; G: glycerol; AA: arachidonic acid; ETA: ethanolamine; AC: adenylyl cyclase; MAPK: mitogen-activated protein kinase; TNFɑ: tumour necrosis factor ɑ; ILs: interleukins; PPAR: peroxisome proliferator-activated receptor; GIRK: G-protein-coupled inwardly rectifying potassium channels. Adapted from Abate et al. (2021) [26]. Created using BioRender.com.

Both cannabinoid receptors are orthosterically modulated by the phytocannabinoid THC and the endocannabinoids (eCBs), anandamide (AEA) and 2-arachidonoylglycerol (2-AG), which are endogenously produced lipid signalling molecules (Figure 2) [19,25,26]. The activation of CB1 inhibits adenylyl cyclase, voltage-gated calcium channels, and cyclic AMP signalling. Receptor activation also promotes mitogen-activated protein kinases (MAPKs) and G-protein coupled inwardly rectifying potassium channels (GIRKs) activity [19,27]. CB1 receptor activation by exogenous THC and the retrograde synaptic movement of eCBs can inhibit both excitatory and inhibitory neurotransmitter release from glutamatergic and GABAergic neurons, respectively [2]. eCB signalling is normally terminated by the hydrolysis of the arachidonic group from 2-AG and AEA. The hydrolysis of 2-AG is primarily carried out by presynaptic monoacylglycerol lipase (MAGL) in the CNS, while postsynaptic fatty acid amino hydrolase (FAAH) primarily breaks down AEA [19,28,29,30]. CBD interacts with the ECS separately, whereby it decreases CB1 receptor activation induced by THC and eCBs and promotes the inverse agonism of CB1 through negative allosteric modulation [19]. CBD has been shown to exhibit antagonistic-like behaviour against synthetic and natural cannabinoids and can also inhibit the psychoactive nature of THC. Multiple studies have confirmed that CBD exhibits a very low affinity for both cannabinoid receptors compared to THC and eCBs [15,19,31,32]. CBD has also been suggested to inhibit eCB uptake and degradation, resulting in prolonged eCB signalling [33]. This occurs through inhibiting FAAH and fatty acid binding protein (FABP) levels and the activity and inactivation of MAGL, thereby limiting hydrolysis and the reuptake of AEA and 2-AG, respectively [15,33,34,35,36].

### 2.2. Pharmacokinetics of Cannabidiol

The pharmacokinetic profile of CBD and its metabolic processes are similar to that of THC, its psychoactive counterpart [15,37]. CBD generally acts in a dose- and delivery-dependent manner [38]. Time to peak drug concentration (T_max_) ranges between 0 and 4 h, while the peak drug concentration (C_max_) is dose-dependent, achieved more quickly through aerosol administration compared to oral, and increased during postprandial states [38]. CBD exhibits low toxicity and low oral bioavailability, ranging from 13 to 19% [15,18]. Intravenously administered CBD is distributed rapidly and can easily pass through the blood–brain barrier (BBB) due to its lipophilic nature [15,37]. CBD is primarily excreted in the urine after several metabolic steps including hydroxylations, oxidations, conjugations, and epoxidations [15,18,39]. A systematic review by Millar et al. focusing on the pharmacokinetics of CBD in humans reported varying half-lives depending on route of administration, with a half-life of 1.4–10.9 h after oral mucosal spray, 24 h post intravenous injection, 31 h after inhalation, and 2–5 days after chronic oral administration [38]. Since these pharmacokinetic differences can significantly impact the amount of CBD reaching the brain, well-designed animal model studies and human trials that take the route of administration and bioavailability into account are needed to evaluate the therapeutic potential of CBD in neurodegenerative conditions like AD.

## 3. Alzheimer’s Disease Pathology

AD is a progressive neurodegenerative disease and the most prevalent form of dementia, accounting for approximately 60–80% of cases [3,4]. Clinical signs of AD include progressive cognitive decline, memory and speech impairment, apathy, depression, mobility issues, and behavioural changes [3,4,5]. These symptomatic manifestations are the result of the neuropathological features of the disease, including neuronal and synaptic loss and the aggregation of extracellular Aβ plaques and intracellular NFTs [3,4,5]. The amyloid hypothesis proposes that the onset and advancement of AD results from an increased abnormal accumulation of Aβ. This occurs through the sequential processing of amyloid precursor protein (APP) by beta- and gamma-secretases, and the subsequent synthesis and accumulation of Aβ oligomers and fibrils, resulting in plaque aggregation [3,4]. Conversely, the tau hypothesis describes the abnormal hyperphosphorylation of tau which results in the formation of NFTs as the main contributor to AD pathogenesis [3,4]. These pathophysiological changes elevate levels of neuroinflammation and oxidative stress, disrupt neuronal synaptic transmission, and induce cellular toxicity [3,4]. Although a highly debated topic, the onset and progression of AD are highly complex and multifactorial, not only directly induced by the two pathophysiological hallmarks, Aβ-plaques and NFTs, but also from several genetic, environmental, and age-related factors. Unfortunately, treatment strategies for AD are limited. While some pharmacological agents only target symptoms after the clinical onset of AD, others that target pathophysiological hallmarks display low efficacy and a plethora of adverse effects [3,4]. For these reasons, there remains a need for investigations into novel primary and adjunct therapies. This section highlights the role of neuroinflammation and oxidative stress in AD, molecular signalling pathways, and potential areas for therapeutic action.

### 3.1. Neuroinflammation in AD

Neuroinflammation, the inflammatory response of the CNS, is characterized by the activation of microglia and the subsequent activation of the proinflammatory cascade [40]. Neuroinflammation begins with the activation of microglia through the detection of damage/pathogen-associated molecular pattern molecules (DAMPs; PAMPs) via their Toll-like receptors (TLRs) and the subsequent release of pro/anti-inflammatory cytokines and chemokines, which is followed by an increase in BBB permeability and peripheral immune cell recruitment [41]. Initially, this is a defence mechanism for damage localized to the site of injury. It aims to promote repair, remove cell debris, and mediate other key neurological processes including immune conditioning, development, learning, and memory [42,43,44]. Despite this, neuroinflammation can become maladaptive, and depending on the severity and duration, it is capable of inflicting further molecular and structural damage to various brain cell types and regions, which over time could induce widespread global degeneration, atrophy, and behavioural impacts [40,45]. Unfortunately, this is a common hallmark observed across several neuropathological disorders and diseases, including AD [42,45].

In AD, neuroinflammation has been associated with the severity of brain atrophy and cognitive decline, with reported increases in proinflammatory cytokines and chemokines like tumour necrosis factor α (TNFα), monocyte chemoattractant protein-1 (MCP-1) interleukin-1β (IL-1β), interleukin-6 (IL-6), and prostaglandins across human AD brain and in vivo AD models [40,45,46,47,48]. These proinflammatory substrates have been known to exacerbate AD pathology by promoting Aβ plaque and tau NFT formations, and similarly, these AD pathological hallmarks have been additionally responsible for upregulating the proinflammatory cascade, resulting in a positive-feedback-loop-like response promoting more inflammation, degeneration, and atrophy [49,50,51,52]. This section discusses the current understanding of neuroinflammation as a main pathology of AD, highlighting the implication of microglia and the proinflammatory cascade as key players in the development of this neurodegenerative disease.

### 3.2. Role of Microglia and the Proinflammatory Cascade in AD

Microglia, the brain’s most important innate immune cells, are crucial in maintaining homeostasis and immune defence mechanisms in the CNS [53]. They have diverse morphologies and play three essential functions: detecting changes in their environment, maintaining physiological homeostasis, and protecting against harmful stimuli by detecting DAMPS through their surface membrane TLRs [53]. In the presence of a stimulus, microglia produce chemokines and proinflammatory cytokines to remove pathological agents and initiate the repair process [53].

In AD pathology, it is well established that microglia are involved in the progression of neurodegeneration by shifting to a cytotoxic and proinflammatory phenotype; however, early in disease onset, microglia have been observed to support brain health and neuroprotection by migrating to areas of the brain with tau and amyloid aggregates and initiating their phagocytosis and clearance [54]. Microglia maintain Aβ homeostasis and utilize different mechanisms for the clearance of either soluble amyloid monomers or larger, insoluble amyloid fibrils [55]. Microglia internalize soluble Aβ through a macropinocytic mechanism, in which soluble Aβ colocalizes to pinocytic vesicles and is rapidly trafficked into late endolysosomal compartments for degradation [56,57]. The receptor-mediated endocytosis of Aβ fibrils occurs through TLRs, scavenger receptor type-A (SRA), triggering receptor expressed on myeloid cells 2 (TREM2), and lipoprotein receptor-related proteins (LRPs) when complexed with lipoproteins, and microglia can further process these fibrils through phagocytosis or upregulated proteinase activity with insulin-degrading enzyme (IDE) or metalloproteinase-9 (MMP-9) [58,59,60,61]. Similarly, microglia also internalize, phagocytose, and degrade tau through competitive binding against fractalkine/CX3CL1 to the CX3CR1 chemokine receptor located on microglia cell membrane surfaces [62,63].

Despite this, microglia are unable to maintain the homeostasis of accumulating amyloid plaques and tau seeding with increasing age, and consequently, researchers have observed the function of microglia to shift from promoting neuroprotection to inducing neurodegeneration [54]. In AD, microglia encourage Aβ plaque formation, the phosphorylation of tau via the p38α MAPK/glycogen synthase kinase 3 (GSK3) pathway, the decreased clearance of Aβ, the reduced clearance of tau through increased competitive binding and the internalization of CX3CL1, the production of proinflammatory molecules and ROS, the structural and functional dysregulation of the BBB, and cognitive decline [49,64,65,66,67,68,69,70]. With the hypofunction in microglial clearance capabilities and the evasion of the microglial degradation of amyloid and tau, their further aggregation into plaques and NFTs occurs, ultimately inducing a chronic neurodegenerative and neuroinflammatory state [71].

**Figure 3:**
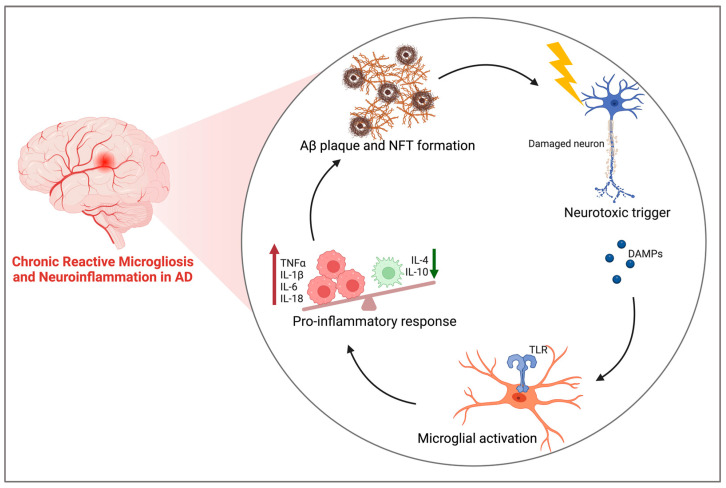
Schematic representation of chronic neuroinflammation in AD and the positive-feedback loop-like response of AD pathological markers promoting reactive microgliosis. Aβ and NFTs induce neuron death triggering the release of DAMPs. These molecules bind to the TLRs on microglia and shift their phenotype to an activated state, initiating the process of microgliosis and subsequent release of proinflammatory cytokines. In chronic neuroinflammation and AD, microgliosis and elevated levels of proinflammatory cytokines then promote the formation of Aβ plaques and NFTs through alterations to crucial molecular signalling pathways and reduced degradation mechanisms [49,53,54,55]. Aβ: amyloid beta; NFT: neurofibrillary tangles, DAMPs: damage-associated molecular patterns; TLR: Toll-like receptor; TNFα: tumour necrosis factor α; IL-: interleukin-. Created using BioRender.com.

Diving further into the positive-feedback-loop-like response by which AD pathology promotes neuroinflammation and vice versa, chronic Aβ deposition leads to microgliosis, which is the activation of microglia to an insult, promoting the upregulated synthesis and release of proinflammatory cytokines, such as IL-1β, TNFα, and IL-6, and the reduced release of anti-inflammatory cytokines like IL-4 and IL-10 (Figure 3) [49]. TNFα, IL-1β, IL-6, and IL-18 have been reported to be upregulated in AD brains and further progress AD pathology by releasing Aβ peptides and attenuating Aβ clearance, thus increasing the opportunity for plaque formation, and by inducing tau hyperphosphorylation and NFT formation through altered p38 MAPK/GSK3 signalling [48,72,73,74,75]. Importantly, microglia produce, secrete, and recruit other secondary molecules, such as prostaglandins and growth factors like brain-derived neurotrophic factor (BDNF), and with AD, these factors are further implicated and linked to the onset and pathology of the disease, highlighting the complexity of neuroinflammation and microgliosis in the progression to a neurodegenerative state [76,77,78,79]. Thus, chronic microglial activation and proinflammatory cytokine release are believed to be both the cause and the effect of exacerbating Aβ and tau aggregates leading to a neurodegenerative AD state.

With our growing understanding of the (1) molecular basis of the neuroinflammatory properties of AD, particularly with positive feedback promoting inflammation and degeneration, and (2) limited therapeutic success with targeting Aβ and immunotherapy/antigen, acetylcholinesterase, and glutamate receptors, there is a need to develop or utilize pre-existing therapies that interrupt these vicious cycles of inflammation and degeneration [10]. As will be discussed later in this review, the anti-inflammatory properties of CBD are encouraging and support its therapeutic applicability in neurodegenerative disease states. Neuroinflammation is also linked to oxidative stress, which is another factor implicated in the development and progression of AD that researchers have explored.

### 3.3. Oxidative Stress

**Figure 4:**
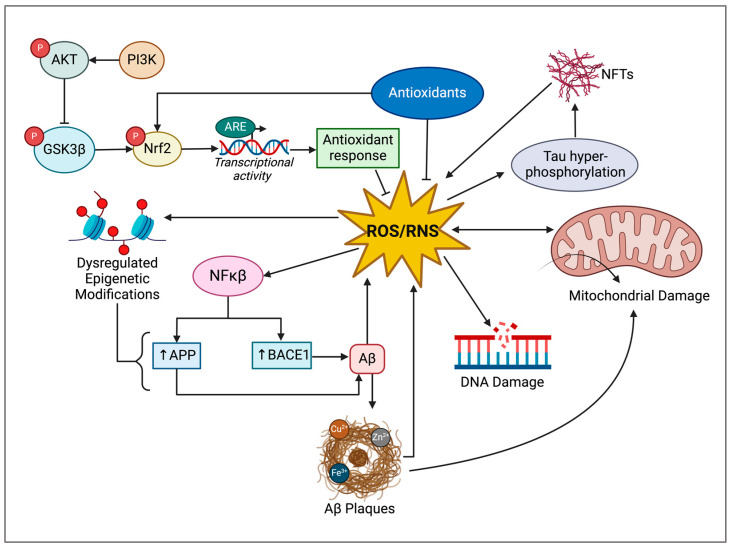
Schematic representation of the role of reactive oxygen and reactive nitrogen species, which are known oxidative stressors, in AD [80,85,86,100,109,110,111,112]. ROS: reactive oxygen species; RNS: reactive nitrogen species; PI3k: phosphoinositide 3-kinase; AKT: protein kinase B; GSK3β: glycogen synthase kinase 3 beta; Nrf2: nuclear factor erythroid 2-related factor 2; ARE: antioxidant response element; NF-κB: nuclear factor κ-light-chain-enhancer of activated B cells APP: amyloid precursor protein; BACE1: beta-secretase 1; Aβ: amyloid-beta; NFTs: neurofibrillary tangles. Created using BioRender.com.

Oxidative stress occurs when there is an imbalance between the production and build-up of toxic ROS and reactive nitrogen species (RNS) and the level and activity of endogenous antioxidants [80]. ROS/RNS are reactive oxygen- or nitrogen-containing molecules that are endogenously produced via mitochondrial oxygen metabolism and nitric oxide (NO) [81]. This imbalance causes damage to biomolecules such as DNA, proteins, and lipids. Common perpetrators of oxidative stress include mitochondrial free radical products such as hydrogen peroxide (H_2_O_2_), hydroxyl radical (^•^OH), superoxide (O_2_^•−^), singlet oxygen (^1^O_2_), and ^•^NO [82]. Although ROS/RNS maintain homeostatic functions in cellular signalling processes at low levels, cellular damage occurs following the excessive accumulation of ROS/RNS [83,84]. To maintain cellular homeostasis, the antioxidant defence system functions to balance the production and elimination of ROS to prevent the development of accelerated aging and disease (Figure 4) [85].

The antioxidant defence system is activated in response to oxidative stress. As a result, endogenous antioxidants are produced and recruited to active sites of oxidative stress. Antioxidants can be divided into either enzymatic or non-enzymatic. Enzymatic antioxidants perform antioxidant functions by scavenging free radicals [86]. This group of antioxidants includes but is not limited to superoxide dismutase (SOD), glutathione peroxidase (GPx), and catalase (CAT) [86]. Non-enzymatic antioxidants can be further divided into polyphenols, vitamins, minerals, and carotenoids [87]. Overall, antioxidants act as scavengers that detect, eliminate, and/or prevent the production of free radicals, inhibit the onset and generation of toxic reactions, and repair damaged biomolecules such as DNA, proteins, and lipids [86].

The transcription factor nuclear factor erythroid 2-related factor 2 (Nrf2) is an important regulator of the endogenous antioxidant response system [88,89]. Nrf2 regulates the basal and stress-induced expression of various antioxidant response element (ARE)-dependent genes to control the physiological and pathophysiological consequences of oxidative stress [88,89]. In the absence of cellular stress, cytoplasmic Nrf2 is bound to its negative regulator Kelch-like ECH-associated protein 1 (Keap1). Dimerization with Keap1 stabilizes Nrf2 [88,89]. This recruits Cullin3 (Cul3) and Ring-box protein 1 (Rbx1), ultimately forming a complex that destines Nrf2 for ubiquitination and eventual proteasomal degradation [88,89]. Because of its short half-life, Nrf2 is frequently degraded and low cellular levels are maintained [90]. Under conditions of stress, including oxidative stress, Nrf2 dissociates from Keap1 and undergoes nuclear translocation [88,89]. In the nucleus, Nrf2 interacts with small Maf proteins and binds to AREs that are found in the promoter region of genes that encode detoxifying and antioxidant enzymes such as heme oxygenase 1 (HO-1), SOD, glutamate-cysteine ligase modifiers and catalytic subunits (GCLM and GCLC, respectively), and GPx [88,89,90].

This dissociation can also be induced by Nrf2 activators such as polyphenolic antioxidant compounds including naringenin and rosmarinic acid [91,92,93,94,95]. Inducers of Nrf2 activation may perform this function via the protein kinase C (PKC)-mediated phosphorylation of Nrf2 at serine-40 [96,97]. Nrf2 is also regulated through the induction of the phosphoinositide 3-kinase (PI3K)/protein kinase B (AKT)/GSK3β pathway [92,93,94,95]. In this pathway, Nrf2 activators induce the activity of PI3K which activates AKT via phosphorylation. AKT then inactivates GSK3β via serine-9 phosphorylation. As a negative regulator of Nrf2, the phosphorylation of GSK3β permits the activity of Nrf2. Both processes induce the phosphorylation and activation of Nrf2, which permits the nuclear translocation of Nrf2 and subsequent transcriptional activity as a function of antioxidant defence. Interestingly, Nrf2 has been reported to be downregulated in AD [94,98]. This has prompted researchers to explore the potential role of compounds such as CBD that may act as antioxidants, Nrf2 activators, and/or regulators of Nrf2 transcriptional activity in response to oxidative stress in AD [99].

### 3.4. Role of Oxidative Stress in AD

Although oxidative stress may exist independently as a contributor to AD, it has also been linked to other hypotheses of AD pathogenesis such as the amyloid and tau hypotheses (Figure 4). The relationship is reciprocal, as oxidative stress can contribute to the phosphorylation of tau and the production and accumulation of Aβ, and in turn, these pathological hallmarks of AD can induce oxidative stress.

Several reports have demonstrated the relationship between oxidative stress and Aβ toxicity in AD [100,101,102,103,104,105,106,107,108]. This supports exploring the possible mechanisms through which oxidative stress induces and/or contributes to the onset and development of AD. One potential mechanism involves metal ions [100,101,102,103,104,105,106,107,108]. Extracellular senile plaques, composed of aggregated Aβ, can contain metal ions such as iron (Fe), copper (Cu), and zinc (Zn) [100,101,102,103,104,105,106,107,108]. When bound to Aβ, these metal ions can induce redox reactions and the generation of ROS [100,101,102,103,104,105,106,107,108]. As a result, newly formed ROS may oxidize Aβ peptides and nearby cellular components such as nucleic acids, proteins, and lipids [100,101,102,103,104,105,106,107,108]. This oxidative process can disrupt membrane integrity via the oxidation of lipids such as cholesterol within the plasma membrane of neurons [109]. Moreover, the ROS and redox-active metal ion-induced oxidation of Aβ hinders the effective clearance of Aβ by LRPs, which further perpetuates Aβ accumulation and the development of AD [110,111]. Additional mechanisms by which oxidative stress promotes Aβ synthesis and accumulation include transcriptional, translational, and epigenetic processes. The activation of stress-related signalling pathways has been reported to induce the transcription of APP, the precursor of Aβ, and BACE1, a crucial enzyme for Aβ production [112]. Some studies have also reported changes in Aβ due to epigenetic modifications such as histone acetylation, DNA methylation, and chromatin remodelling that contribute to AD [112,113,114,115,116,117,118]. More recently, researchers such as Gu et al. have established a relationship between epigenetic changes in Aβ synthesis and oxidative stress following H_2_O_2_ treatment in neuroblastoma cells [118]. These epigenetic changes include a significant increase in histone acetylation and a decline in DNA methylation, resulting in heightened Aβ production following increased APP and BACE1 transcription [118]. Interestingly, Aβ has also been reported to exert toxic cellular effects by stimulating oxidative stress via mitochondrial disruption and interfering with neuronal processes [119,120,121,122]. This ultimately leads to cytoskeleton disruption, synaptic dysfunction, and neuronal apoptosis [121].

Oxidative stress-induced tauopathies have also been investigated as contributors to the pathogenesis and progression of AD (Figure 4). Researchers have recently established a reciprocal relationship between tau pathology and oxidative stress [123]. Potential mechanisms driving these effects include increased tau phosphorylation and mitochondrial dysfunction, driving the increased production of ROS such as H_2_O_2_ [123,124,125,126]. On the other hand, increased levels of oxidative stress result in elevated tau phosphorylation, perpetuating a vicious cycle [123]. This hyperphosphorylation hinders the binding affinity of tau to microtubules, which results in the destabilization of microtubules along with the development of hyperphosphorylated tau oligomers [123,124,125,126]. Following this, hyperphosphorylated tau oligomers may accumulate to form NFTs that trigger neurotoxicity and neuronal cell death [127,128]. Overall, these events contribute to the degradation of microtubule networks that trigger the neurodegeneration observed in AD.

Whether oxidative stress is the cause, or the result of tau aggregation, amyloid toxicity, and other AD-related neurological pathologies remains under debate. What is not debated is the participation of oxidative stress in the pathophysiology of AD, and as such, this represents a promising target for AD therapy. In addition to its antioxidant properties, CBD also reduces neuroinflammation, which, as described previously in this review, is also implicated in AD.

## 4. Cannabidiol in Preclinical Models of Alzheimer’s Disease

As described above, the dysregulation of antioxidant and anti-inflammatory modulators is highly implicated in the onset and progression of AD, including its molecular pathologies and eventual symptomatic outcomes. CBD has been proposed to regulate several aspects of the ECS and has been reported to be capable of combating oxidative stress and neuroinflammation across a variety of aetiologies [1,2,11,12,13]. Recently, a breadth of research has been invested in determining if CBD is capable of protecting against these stressors in several preclinical models of AD. This section will focus on the currently available literature utilizing CBD in models of AD in vitro (Table 1) and in vivo (Table 2), and on their outcomes related to AD-induced oxidative stress and neuroinflammation.

### 4.1. CBD’s Treatment of AD-Related Pathologies

#### 4.1.1. Modulation of Neuroinflammation by CBD

CBD has been reported to beneficially modulate markers of neuroinflammation in preclinical models of AD. Specifically, investigations in vitro have displayed CBD-mediated protection against neuroinflammation, decreased proinflammatory molecules, and increased anti-inflammatory cytokine levels [136,140]. For example, Esposito et al. reported that CBD decreased the levels of proinflammatory cytokines IL-1β and TNFα in primary rat astroglial cultures [136,140]. They also found that CBD decreased p50 and p65 protein expression through selective peroxisome proliferator-activated receptor γ (PPARγ)-dependent nuclear factor κ-light-chain-enhancer of activated B cells (NF-κB) inhibition [136]. Another study recapitulated these findings in differentiated NSC-34 cells, reporting that CBD in combination with cannabigerol (CBG), but not CBG alone, decreased TNFα levels and NF-κB activation while increasing the expression of the anti-inflammatory cytokines IL-10 and IL-37 [140]. These in vitro findings suggest that CBD could provide neuroprotection through enhancing anti-inflammatory pathways and inhibiting proinflammatory cascades that, as discussed above, have an established role in AD.

Several in vivo investigations have also demonstrated significant improvements in AD-related markers of neuroinflammation by way of CBD treatment. BDNF, which can be modulated under conditions of neuroinflammation, is reduced in the brains of individuals with AD, and a study by Kim et al. reported that cannabidiolic acid (CBDA), the precursor of CBD, increased hippocampal BDNF levels in Aβ-treated ICR mice [137]. This was accompanied by the phosphorylation of BDNF’s receptor, tropomyosin receptor kinase B (TrkB), and the activation of TrkB signalling molecules and transcription factors, including cAMP response element-binding protein (CREB) [137]. Low BDNF levels could result from an increased expression of proinflammatory cytokines, and Esposito et al. reported that 7-day intraperitoneal CBD treatment decreased proinflammatory cytokine levels and subsequent IL-1β release [145]. Although Martín-Moreno et al. reported CBD-mediated decreases in IL-6 levels in Aβ-treated C57/B16 mice, in this study, CBD did not alter TNFα levels. Interestingly, a study by Cheng et al. did not identify statistically significant changes in proinflammatory cytokine levels following the chronic oral administration of CBD to a double transgenic mouse model of AD (AβPPxPS1) [148]. Similarly, Watt et al. found that a 3-week intraperitoneal injection of CBD in the same transgenic mice did not reduce markers of neuroinflammation; however, hippocampal levels of Aβ were reduced [149]. In contrast, in non-AD models of neuroinflammation, two studies demonstrated that CBD treatment decreased the levels of proinflammatory cytokines [152,153]. CBD also increased the levels of the anti-inflammatory cytokine IL-10 and inhibited the expression of the NLR family pyrin domain containing 3 (NLRP3) inflammasome [153]. Although these investigations did not utilize models of AD pathology-induced neuroinflammation, the specific markers of interest are highly relevant to AD pathology.

#### 4.1.2. Modulation of Oxidative Stress by CBD

CBD has also been reported to have antioxidant properties capable of protecting against a variety of stressors and molecular regulators of oxidation. Several in vitro models have determined that CBD can decrease the accumulation of ROS and RNS, modulate antioxidant pathways, and decrease the expression of enzymatic generators of oxidative molecules. For example, CBD was reported to protect against tert-butyl hydroperoxide (t-BHP)-, Aβ-, and H_2_O_2_-induced oxidative stress in studies employing multiple cell lines, including rat PC12 and human SH-SY5Y and primary astrocytes [131,132,134,135]. These results were exhibited through general decreases in ROS accumulation and improvements in cell viability with CBD treatment [131,132,134,135]. CBD was also shown to prevent Aβ-generated increases in inducible nitric oxide synthase (iNOS) and the associated release of NO and nitrite in rat PC12 and astroglial cells and mouse-derived NSC-34 cells [130,136,140]. In addition to CBD’s direct antioxidant properties, this phytocannabinoid has also been shown to regulate specific molecular markers of the oxidative stress response. Esposito et al. reported that CBD decreases iNOS expression and nitrite release by inhibiting phosphorylated p38 MAPK and NF-κB activation [130]. Another study conducted by Mammana et al. showed that a combination of CBD and CBG, but not CBG alone, increased the level of Nrf2, which is highly implicated in the endogenous antioxidant response system [140].

In vivo models of AD have also shown promise for the antioxidant potential of CBD. Esposito et al. evaluated the effects of CBD on Aβ-induced stress in a mouse model of AD, reporting decreases in iNOS expression and NO release after a 7-day intraperitoneal administration of CBD [145]. These results were more recently replicated and expanded on by the same investigators, who demonstrated the CBD-induced decreases in iNOS were modulated through PPARγ dependent NF-κB inhibition, with associated decreases in reactive gliosis and improved hippocampal neuron survival in rats [136]. Additionally, AD-like models of Caenorhabditis elgans (*C. elgans*) confirmed many of the previous in vitro findings [135,154,155]. These investigations showed that CBD not only decreased total levels of ROS but inhibited ROS accumulation independent of antioxidative genes and positively modulated the Nrf2 pathway through NF-κB inhibition [135,154,155].

Together, these results demonstrate the ability of CBD to modulate markers of AD pathology, target oxidative stress by reducing the production of ROS and enhancing antioxidant capacity, and protect against neuroinflammation by positively regulating anti-inflammatory modulators and inhibiting proinflammatory cytokine levels. They also highlight important differences that can impact the effects of CBD in vivo. In addition to the different models of oxidative stress and neuroinflammation used in these studies, the routes of administration, dose, and duration of CBD treatment differed. As discussed previously, this can impact the bioavailability of CBD as well as the levels of CBD within the brain. Although some differences were reported, these findings prompt interest in the AD-related anti-inflammatory and antioxidant properties of CBD, while also demonstrating the need for further AD-specific investigations with human-physiologically relevant modes of drug administration and dosing.

## 5. Cannabidiol as a Potential Treatment for Alzheimer’s Disease

The effectiveness of CBD as a potential treatment for AD is centred around its ability to beneficially modulate anti-inflammatory and antioxidative molecular pathways and transcription factors. As described above, CBD has been reported to protect cell cultures, *C. elgans*, and rodent models from AD-like oxidative and neuroinflammatory stressors [130,131,132,134,135,136,137,140,145,146,148,149,152,153,154,155]. CBD was also reported to increase the levels of anti-inflammatory cytokines, such as IL10 and IL37, while conversely decreasing the expression of proinflammatory cytokines TNFα, IL6, and IL-1β [136,140,145,146,148,149,152,153,154,155]. These regulatory changes in cytokine expression are associated with inhibiting NF-κB activation, a crucial regulator of inflammation [130,136,140,154]. CBD can also inhibit iNOS expression and the subsequent release of oxidative molecules, such as NO and nitrite, while promoting increases in the Nrf2 endogenous antioxidant response system [130,136,140,145,154].

Recent investigations have also demonstrated that CBD is not only able to modulate molecular markers and regulators of inflammation and oxidation, but can also reduce the levels of Aβ and phosphorylated tau [133,137,138,139,142]. In vitro studies have demonstrated that treatment with CBD can increase transient receptor potential cation channel subfamily V member 2 (TRPV2)-activation-dependent microglial Aβ phagocytosis, inhibit amyloid toxicity, and ultimately decrease the total levels of Aβ aggregation in mouse cortical neurons and microglial cells and human MC65 cells [137,138,139]. In addition, Scuderi et al. reported that in APP+-expressing SH-SY5Y cells, CBD decreased APP and Aβ peptide levels by increasing APP ubiquitination through PPARγ activation [133]. Alali et al. showed that CBD decreased tau aggregation rate and levels in *Escherichia coli* (*E. coli*)-expressing human His-tagged tau protein delivered through the pET-21(+) expression vector system [142]. Interestingly, a study by Kim et al., utilizing both primary mouse cortical neurons and Aβ-treated female ICR mice, displayed CBDA’s ability to decrease the levels of pathophysiologically relevant markers of AD, namely, APP, Aβ and hyperphosphorylated tau [137].

The modulation of these quantifiable molecular markers of AD by CBD and its precursor CBDA were further translated into AD-relevant changes in cell viability in vitro, and physiological and cognitive-behavioural changes in vivo [133,134,135,136,137,138,139,141,143,144,146,148,149,150,152,154]. Multiple investigations revealed that CBD did not display neurotoxicity at physiologically relevant doses, while also increasing the viability of SH-SY5Y cells and mouse cortical neurons and striatal-derived STHdh^Q7/Q7^ cells compared to cells treated only with neurotoxic compounds, such as Aβ_1–42_ and t-BHP [133,134,137,141]. Patel et al. also showed that STHdh^Q7/Q7^ cells pre-treated with CBD exhibited increased pro-survival markers of the unfolded protein response (UPR) at the mRNA and protein level, while decreasing mRNA expression of pro-apoptotic genes Bcl-2-like protein 11 (BIM) and caspase-12 [141]. Although some studies reported that the treatment of rodent models of AD with CBD alone had no effect on AD-related pathologies or cognition, many reported opposite findings [147,148]. Esposito et al. demonstrated that CBD decreased reactive gliosis and increased neuron survival in the hippocampus of rats treated with Aβ_1–42_ [136]. At the same time, de Paula Faria et al. reported CBD-induced decreases in brain glucose hypometabolism and weight loss in streptozotocin (STZ)-treated rats [144]. Although the STZ treatment time was not long enough to induce Aβ pathology, the memory deficits in this model, demonstrated through object recognition, were attenuated by CBD [145]. Similarly, multiple investigations have demonstrated that CBD treatment positively affects memory and cognition in rodent models of AD [137,143,144,146,148,149,150,151,152]. For example, four studies utilizing a double transgenic mouse model of AD (AβPP × PS1 mice), reported that CBD restored impairments in object recognition, spatial learning, and social recognition memory [148,149,150,151].

The studies described above highlight CBD’s broad range of potential therapeutic effects. In vitro studies consistently describe multiple therapeutically relevant effects of CBD, including reducing markers of AD pathology, inflammation, and oxidative stress. Multiple in vivo studies support the anti-inflammatory, antioxidant, and neuroprotective properties of CBD and, in addition, have demonstrated CBD-mediated improvements in cognitive performance. The preclinical results summarized in Table 1 and Table 2 suggest that CBD has the potential to delay the pathophysiological progression and cognitive-behavioural changes that occur in humans with AD. Since currently approved treatments for AD only provide limited symptomatic benefits, exploring the therapeutic efficacy of compounds like CBD, that have the potential to target multiple pathological mechanisms, is crucial. Further investigation of CBD’s action in preclinical models and effects in human clinical trials in individuals with AD is needed.

## 6. Future Directions and Limitations of Cannabidiol in Alzheimer’s Disease

Although preclinical studies support CBD’s promise as a potential treatment for AD, there are several limitations and areas for future research to address. First, despite encouraging results from preclinical studies, to date, no human randomized control trials (RCTs) have investigated the efficacy and safety of CBD in treating AD [2,11,26,156,157,158,159,160,161,162,163,164,165,166,167,168]. Second, the optimal dosage, administration route, formulation, and treatment duration of CBD for achieving effective management of AD is unclear, and determining this remains challenging [38,158,159,160,162,164,165,169,170,171]. Individual variability in responses to CBD and factors like age, sex, and genetic predisposition complicate establishing optimal treatment protocols [2,159,166]. The complexity of CBD’s pharmacokinetic profiles and variations in bioavailability among different administration routes, distribution patterns within the body, and its safety profile are all factors that need to be further investigated [37,170]. Currently, there are no standardized dosing guidelines and formulations for CBD across studies, making comparisons difficult. In the context of AD, symptom severity and disease stage can impact optimal dosing regimens [172]. For instance, higher doses or a longer duration of CBD treatment may be needed to target severe cognitive symptoms. Third, given the multifactorial nature of AD pathology, CBD may need to be considered as an adjuvant therapy, complementing currently approved first-line treatments such as acetylcholinesterase inhibitors [2]. Next, despite CBD lacking psychoactive effects, the stigma and misconception associated with cannabis and its derivatives persist and may hinder patient acceptance of CBD-based therapies for AD [14,159,173,174,175]. Additionally, there are legal and regulatory restrictions that exist in some countries, creating barriers to CBD research and access [156,157,159,160,161,163]. Lastly, CBD is a relatively recent topic of investigation in the context of AD, and our understanding of its mechanisms of action, therapeutic potential, long-term effects, and drug–drug interactions is still evolving [2,11,26,156,157,158,159,160,161,162,163,164,165,166,167,168,171].

To address these limitations, future investigations should focus on conducting comparative studies to evaluate different CBD doses, administration routes, formulations, treatment durations, and administration timing and frequency. These are necessary for both in vivo preclinical and human studies. In addition, for human studies, factors such as age, sex, race, disease severity, and potential co-treatment benefits and drug–drug interactions with medications commonly used by AD patients should be considered. Following the completion of these studies, the next steps would involve conducting well-designed, large-scale, double-blinded RCTs with clinically quantifiable endpoints to validate CBD’s therapeutic effects in humans with AD. Long-term follow-up with participants is essential to document adverse effects, characterize safety profiles, and assess true efficacy. To address misconceptions and reduce the stigma associated with CBD, educational campaigns can be conducted to provide accurate information, promote acceptance, and educate patients, caregivers, and the general public. Improving communication between patients, caregivers, and healthcare professionals is crucial to avoid misconceptions and ensure informed decision-making regarding CBD treatment. Addressing these limitations and conducting further research will be crucial for advancing our understanding of CBD and developing effective CBD-based treatment for AD.

## 7. Conclusions

Preclinical studies support the potential for CBD to be used as a treatment for combatting neuroinflammation and oxidative stress in AD. This is demonstrated by CBD’s ability to modulate pro/anti-inflammatory cytokines, markers of oxidative stress, molecular pathways in the endogenous antioxidant response system, and proinflammatory transcription factors, and its associated positive changes in pathological hallmarks of AD in vitro and in vivo and improvement in cognitive-behavioural outcomes in rodent models of AD. These benefits, along with widespread consensus on its anti-inflammatory and antioxidant properties, make CBD an interesting future candidate for therapeutic use in AD.

Although preclinical findings are promising, human clinical trials utilizing CBD in the context of AD are lacking. As current evidence supports the therapeutic benefits of CBD as a modulator of neuroinflammation and oxidative stress in AD, there is a need for further preclinical research and RCTs investigating the effects of CBD. Future research should focus on determining a physiologically relevant route of administration and optimal dosing regimen in preclinical studies and humans in order to gain a better understanding of CBD’s mechanism of action and determine the efficacy of CBD in human clinical trials of AD.

## Figures and Tables

**Table 1:** In vitro models of AD using CBD.

First Author and Year	Model	Treatment (CBD)	Primary Outcome Measures	Main Results
Esposito 2006 [129]	PC12 cells (NGF diff.)	CBD 15 min pre-treatment (10^−7^–10^−5^ M); Aβ_1–42_ (1 μg/mL) 24 h	Tau hyperphosphorylation modulated through the Wnt/β-catenin pathway	CBD: ↓ Aβ (1–42)-induced p-GSK-3β; ↑ β-catenin; ↓ p-tau
Esposito 2006 [130]	PC12 cells (NGF-diff.)	CBD 15 min pre-treatment (10^−6^–10^−4^ M); Aβ_1–42_ (1 μg/mL) 24-hour	iNOS expression and NO production through p38 MAPK and NF-κB action	CBD: ↓ nitrite and iNOS expression; ↓ p-p38 MAPK; ↓ NF-κB activation
Iuvone 2004 [131]	PC12 cells	CBD (10^−7^–10^−4^ M) and Aβ_1–42_ (1 μg/mL)	Neuroprotection against Aβ-induced neurotoxicity by modulating ROS levels, lipoperoxidation, and apoptosis	CBD: ↓ cell death; ↓ ROS accumulation and lipid peroxidation; ↑ procaspase–total caspase 3
Harvey 2012 [132]	PC12 and SH-SY5Y cells	*PC12:* CBD (1 or 10 μM) & Aβ_1–40_ or H_2_O_2_ or *t*-BHP; *SH-SY5Y*: CBD (0.01–10 μM) and H_2_O_2_ or *t*-BHP	Oxidative stress and Aβ-induced neurotoxicity: CBD compared to known antioxidants and anandamide	No effect of CBD against H_2_O_2_ or Aβ_1–40_ in PC12 cell viability; CBD (10 μM) ↑ cell viability against *t*-BHP in both cell lines
Scuderi 2014 [133]	SH-SY5Y^APP+^ cells	CBD (10^−9^–10^−6^ M)	Modulation of APP in APP-overexpressing cells and the involvement of PPARγ	CBD: ↓ APP and Aβ peptide levels and ↑ APP ubiquitination through PPARγ activation; ↑ cell viability
Raja 2020 [134]	SH-SY5Y cells (RA-diff.)	CBD (0.01–74 μg/mL) and Aβ_1–42_ (10 μM) or H_2_O_2_ (100 μM)	H_2_O_2_-induced oxidative stress and Aβ_1–42_-Cu(II) simulated AD-like oxidative stress	CBD: inhibits H_2_O_2_-induced ROS (IC_50_ = 42.7 μg/mL); displays no neurotoxicity (<1 μg/mL)
Wang 2023 [135]	1’ human astrocytes	CBD (2.5 μM); Aβ_1–42_ (2 μM)	Aβ-induced cellular senescence and apoptosis with the involvement of Parkin	CBD: ↓ Aβ-induced astrocyte senescence and rescues Aβ-induced mitophagy deficits; ↓ mitochondrial ROS
Esposito 2011 [136]	1’ rat astroglial cultures	CBD (10^−9^–10^−7^ M); Aβ_1–42_ (1 μg/mL)	Role of PPARγ receptor activity in CBD-mediated neuroprotection	CBD: ↓ release of NO, IL-1β, TNF-α, and S100B; ↓ Aβ-induced iNOS, GFAP, and S100B protein; ↓ p50 and p65 through selective PPARγ-dependent NF-κB inhibition
Kim 2023 [137]	1’ mouse cortical neurons	CBDA (3 & 6 μM); Aβ_1–42_ (5 μM)	Aβ-induced AD-like characteristics	CBDA: ↓ Aβ and p-tau levels; alleviated calcium dysfunction; ↑ cell viability
Yang 2022 [138]	1’ mouse microglial cultures	CBD (5 μM); Aβ_1–42_ (1 μg/mL)	Effects of CBD on TRPV2 expression and microglial Aβ phagocytosis	CBD: ↑ TRPV2-activation dependent microglial Aβ phagocytosis, mitochondrial function, and ATP production
Schubert 2019 [139]	MC65 cells	CBD (0.1 μM)	Neuroprotective capacity of 11 cannabinoids against Aβ-induced neurotoxicity and aggregation	CBD: inhibited amyloid toxicity and ↑ degradation and removal of Aβ
Mammana 2019 [140]	NSC-34 (serum deprived and RA-diff.)	CBD and/or CBG (2.5, 5, 10, 20, 40, and 80 μM)	Effect of CBG and CBD, alone and in combination, on neuroinflammation through cytokine, NF-κB, and Nrf2 involvement	CBG w/CBD: ↓ neuroinflammation (2.5 and 5 μM); ↓ TNF-α levels and NF-κB activation; ↑ IL10 and IL37 expression (5 μM); ↓ iNOS and ↑ Nrf2 levels (5 μM)
Patel 2023 [141]	STHdh^Q7/Q7^ cells	CBD (1 μM) pre-treatment, co-treatment, and post-treatment w/TG	Neuroprotection against TG-induced ER stress through the modulation of pro-survival and pro-apoptotic factors	CBD pre-treatment: ↑ cell-viability; ↑ pro-survival UPR mRNA expression (GRP78, MANF, and BCL-2) and protein levels (GRP78); ↓ pro-apoptotic mRNA expression (BIM and Caspase-12)
Alali 2021 [142]	*E. coli* BL21	CBD (0, 10, 20, and 40 μM) in tau protein solution (20 μM)	Aggregation of recombinant human His-tagged tau protein expressed through pET-21a (+) vector	CBD: ↓ heparin-induced tau protein aggregation rate and levels

**Table 2:** In vivo models of AD using CBD.

First Author and Year	Model	Treatment (CBD)	Primary Outcome Measures	Main Results
Esposito 2011 [136]	Aβ-treated male Sprague Dawley rats	15-day intraperitoneal CBD (10 mg/kg); hippocampal Aβ_1–42_ (1 μg/mL)	Involvement of PPARγ in the neuroprotective effects of CBD following intrahippocampal injection of Aβ (1–42)	CBD: ↓ iNOS, GFAP, and S100B through PPARγ-dependent inhibition of NF-κB; ↓ reactive gliosis and ↑ neuron survival in rat hippocampus
Fagherazzi 2012 [143]	Iron-induced model of ND in male Wistar rats	Intraperitoneal CBD (5 and 10 mg/kg); oral Fe^2+^ (30 mg/kg; 3 days)	Effects of CBD in iron overload-induced memory impaired rats	CBD: acute highest dose ↓ memory impairment w/chronic treatment; ↑ recognition memory w/chronic treatment; no effect on memory in CBD-treated control rats
de Paula Faria 2022 [144]	STZ-induced AD male Wistar rats	7-day intraperitoneal CBD (20 mg/kg); STZ (3 mg/kg)	Effect of CBD on brain glucose metabolism and cognitive function measured through PET imaging	CBD: ↓ brain glucose hypometabolism and memory damage; ↓ total weight loss
Esposito 2007 [145]	Aβ-treated C57BL/6J mice	7-day intraperitoneal CBD (2.5 or 10 mg/kg); hippocampal Aβ_1–42_ (10 ng)	Anti-inflammatory and antioxidant effects of CBD in mice with Aβ-induced neuroinflammation	CBD: ↓ GFAP mRNA and protein expression; ↓ iNOS and IL-1β protein levels, and related NO and IL-1β release
Kim 2023 [137]	Aβ-treated female ICR mice	Hippocampal CBDA (6 μM); hippocampal Aβ_1–42_ (3 μM)	Effect of CBDA on Aβ-induced AD-like symptoms and pathology	CBDA: ↓ hippocampal Aβ and p-tau levels; ↑ cognitive function; ↑ hippocampal BDNF, p-TrkB, and p-CREB levels
Martín-Moreno 2011 [146]	Aβ-treated C57/Bl6 mice	Intraperitoneal CBD (20 mg/kg); intraventricular Aβ_1–40_ (2.5 μg)	Effects of CBD compared to other cannabinoids in Aβ-induced memory-deficits and inflammatory cytokine expression	CBD: ↓ Aβ-induced cognitive impairments; ↓ IL6 expression but no effects on TNF-α
Arnanz 2023 [147]	5xFAD mice	28-day CBD (0.273 mg/kg) and CBD:THC (0.273:0.205 mg/kg)	Neuroprotective effects of chronic low-dose cannabinoid treatment in 5xFAD mice	CBD:THC: ↑ spatial memory All treatments: ↑ cortical insoluble Aβ
Cheng 2014 [148]	Male AβPP × PS1 mice	8-month oral CBD (20 mg/kg)	Effect of chronic CBD treatment on memory, anxiety, Aβ load, oxidative damage, cholesterol, and neuroinflammation in transgenic model of AD	CBD: ↓ social recognition deficits; no effect on anxiety, learning, Aβ load, or oxidative damage; ↑ cholesterol in WT mice; non-sig. ↓ in cytokines
Watt 2020 [149]	Male AβPP × PS1 mice	3-week intraperitoneal CBD (50 mg/kg)	Behavioural and anti-inflammatory effects of chronic CBD administration in transgenic model of AD	CBD: restored social recognition memory and spatial learning deficits; ↓ Aβ in hippocampus; no effect on neuro-inflammation or PPARγ
Coles 2020 [150]	Female AβPP × PS1 mice	Chronic intraperitoneal CBD (5 mg/kg)	Behavioural effects of medium-dose chronic CBD treatment administration in transgenic model of AD	CBD: restored object recognition and spatial learning deficits
Cheng 2014 [151]	Male AβPP × PS1 mice	3-week intraperitoneal CBD (20 mg/kg)	Behavioural effects of chronic CBD treatment administration in transgenic model of AD	CBD: restored novel object recognition and social recognition impairments; no changes to anxiety-related behaviours
Garcìa-Baos 2021 [152]	PLAE C57BL/6 mice	CBD (20 mg/kg; 10 days)	Anti-inflammatory effects of CBD in a mouse model of FASD	CBD: ↓ cognitive deficits; ↓ PLAE-induced increases in TNF-α and IL6 in hippocampus
Wang 2022 [153]	MPTP-induced PD C57BL/6 male mice	14-day oral CBD (100 mg/kg); intraperitoneal MPTP (30 mg/kg)	Neuroprotective effects of CBD on MPTP-induced PD mice	CBD: ↓ TNF-α, IL6 and IL-1β; ↑ IL-10; ↓ expression of NLRP3, caspase-1, and IL-1β inflammasome
Frandsen 2022 [154]	*C. elgans* transgenic model of AD	CBD (100 μM; 24 h)	Modulation of the glyoxalase pathway and the involvement of Nrf2 and NF-κB in Aβ-expressing *C. elgans*	CBD: ↑ survival; ↓ Aβ fluorescence; ↑ Nrf2 mediated protein levels through NF-κB inhibition
Zhang 2022 [155]	Aβ_1–42_-treated *C. elgans*	CBD (100 μM)	Effect of CBD on Aβ aggregation and Aβ-induced AD-like characteristics in *C. elgans*	CBD: ↓ Aβ aggregation; ↓ ROS independent of antioxidative genes
Wang 2023 [135]	*C. elgans* transgenic model of AD	CBD (5 μM)	Effects of CBD treatment on lifespan, ROS, and pumping rate in Aβ-expressing *C. elgans*	CBD: ↑ lifespan, ↓ ROS and restored pumping rate

## Data Availability

Not applicable.
